# Embryological Characteristics of Human Oocytes With Agar-Like Zona Pellucida and Its Clinical Treatment Strategy

**DOI:** 10.3389/fendo.2022.859361

**Published:** 2022-06-23

**Authors:** Dandan Yang, Han Yang, Bo Yang, Kaijuan Wang, Qi Zhu, Jing Wang, Fangfang Ding, Bihua Rao, Rufeng Xue, Jing Peng, Qiushuang Wang, Yunxia Cao, Weiwei Zou, Beili Chen, Zhiguo Zhang

**Affiliations:** ^1^ Reproductive Medicine Center, Department of Obstetrics and Gynecology, The First Affiliated Hospital of Anhui Medical University, Hefei, China; ^2^ National Health Commission Key Laboratory of Study on Abnormal Gametes and Reproductive Tract (Anhui Medical University), Hefei, China; ^3^ School of Biomedical Engineering, Anhui Medical University, Hefei, China

**Keywords:** human oocyte, agar-like ZP, R-ICSI/ICSI, embryo development, clinical outcomes

## Abstract

Zona pellucida (ZP) abnormalities are the cause of low fertility or infertility, agar-like ZP is more common in abnormal ZP. The purpose of this exploration is to systematically analyze the fertilization competence of agar-like ZP oocytes, the development characteristics of subsequent embryos as well as the results of embryo transfer, aiming to explore effective clinical treatment strategies. A total of 58 patients with agar-like ZP were set as the case group and the control group involved 3866 patients, in which the patients’ oocytes presented normal ZP. BMI, basal hormone levels, and hormone levels were similar in both groups. The case patients suffered significantly longer infertility years than control (p<0.05), and most patients were diagnosed with pelvic inflammatory diseases. A distinct difference was observed in the structure of oocyte corona cumulus complexes between the two groups. The embryo development parameters, which include the rates of cleavage, high-quality embryo, blastocyst, and high-quality blastocyst in the case group were greatly lower than that in the control group (p<0.05). The rates of cumulative clinical pregnancy and live birth were comparable between the two groups. In the subsequent follow-up, thirty-four of the 58 patients receiving intracytoplasmic single sperm injection (ICSI) or early rescue ICSI (R-ICSI) treatment successfully gave birth to babies, and all of the newborns were with no neonatal defects. In addition, the fertilization rate of the R-ICSI group was significantly lower than that of the ICSI group (p<0.05). The occurrence of agar-like ZP impairs the development competence of human oocytes, however, the human oocytes with agar-like ZP can develop into healthy offspring, and an ICSI regimen is the optimal treatment strategy for them.

## Introduction

*In vitro* fertilization-embryo transfer (IVF-ET) technology is currently one of the most widely-used assisted reproductive technologies (ARTs) (1), and various abnormal morphology of oocytes are observed with the increasing use of ART. Intracellular malformations include milky or dark cytoplasm (2), while extracellular defects involve abnormal shapes of zona pellucida (ZP), perivitelline space, and the first polar body, etc.

The ZP is an extracellular coat that surrounds the mammalian oocyte and early embryo. The ZP of human oocytes consists of four glycoproteins designated ZP1, ZP2, ZP3, and ZP4, which are synthesized and secreted by the oocyte (3, 4). These glycoproteins gather around the developing oocyte to form fibers, in which ZP2 and ZP3 assemble into heterodimer repeating units in the extracellular space and are cross-linked by ZP1 or ZP4 (5). The binding of capacitated acrosome-intact spermatozoa to ZP3 and ZP4 glycoproteins in the ZP is a prerequisite for fertilization (6).

The ZP plays a crucial role in follicular development, sperm-ovum bonding, acrosomal reaction induction, prevention of polyspermy, and oviductal transportation. The complete structure guarantees its normal function to achieve normal pregnancy (7). ZP abnormalities are the cause of low fertility or infertility (8), and their incidence accounts for 2-5% in all oocytes (9). Several abnormal ZP has been reported, including zona-free, thickness abnormality, irregular shape, and perivitelline space abnormality. Among them, the oocytes without ZP can become pregnant through intracytoplasmic single sperm injection (ICSI) insemination followed by embryo culture *in vitro* and embryo transfer; the thickness of ZP is negatively correlated with the embryo implantation rate, and the normal fertilization rate is significantly reduced in the oocytes with a dark and irregular ZP, eventually affecting the pregnant outcome (10–13). Therefore, in clinical treatment, morphological analysis for the ZP is an important way to assess the quality of oocytes (14).

Agar-like ZP is more common in abnormal ZP. After denudation for the oocyte, the ZP appears compact and bright under an inverted microscope, showing a uniform agar-like structure, with a complete or partial absence of the perivitelline space (12). Human oocytes with agar-like ZP are not capable of natural fertilization because sperm cannot penetrate the ZP into the cytoplasm. Therefore, such patients undergoing conventional IVF-ET treatment will first suffer IVF fertilization failure. Although ICSI can reverse the fertilization failure of oocytes, ICSI insemination can only be performed after the IVF fertilization failure (15) because no specific clinical characteristics can help to diagnose the occurrence of the abnormal structure before ART therapy cycles. At present, short-term fertilization technology is widely developed in China. Therefore, the development of oocytes following early rescue ICSI (R-ICSI) insemination is a key node in the treatment of such patients.

The literature involved in this abnormal ZP is relatively scarce, so one of the purposes of this exploration is to enrich this information. The present study reviewed the clinical data of 68 Ovum pick-up (OPU) cycles (58 patients), in which the oocytes retrieved were all with agar-like ZP, and the fertilization competence of oocytes, the development characteristics of subsequent embryos as well as the results of clinical treatment were systematically analyzed aiming to explore effective clinical treatment strategies.

## Materials and Methods

### Ethics Statement

The present study was initiated and conducted after the Committee of Medical Ethics approval from Anhui Medical University (Hefei, China; Approval No. 20170049).

### Clinical Data Collection

Clinical data of the infertile patients undergoing IVF/ICSI treatment in the First Affiliated Hospital of Anhui Medical University from May 2015 to October 2020 were collected. A total of 58 patients with agar-like ZP (68 OPU cycles) were set as the case group and the control group involved 3866 patients (4156 OPU cycles), in which the patients’ oocytes presented normal ZP. In this study, Patients with chromosomal abnormalities, pelvic tumors, immune system diseases, and severe oligozoospermia of the partner’s sperm were excluded. The detailed baseline information from the women was shown in **Table 1**.

**Table 1:** Basic clinical information of patients.

Index	Case Group	Control Group	P-value
Total OPU cycles (n)	68	4156	–
No. of patients (n)	58	3866	–
Age (years)	30.16 ± 3.92	31.44 ± 5.19	0.043
Infertility duration (years)	4.41 ± 3.09	3.52 ± 2.79	0.015
Primary infertility rate (%)	72.41 (42/58)	49.23(2046/4156)	<0.005
BMI (kg/m^2^)	22.20 ± 3.61	22.72 ± 3.45	0.246
FSH (IU/L)	7.50 ± 2.59	7.55 ± 2.92	0.894
LH (IU/L)	4.97 ± 2.87	5.53 ± 3.41	0.224
E2 (pmol/L)	187.3 ± 186.8	174.6 ± 194.9	0.623
P (nmol/L)	2.98 ± 5.90	2.42 ± 4.06	0.334
PRL (ng/ml)	24.83 ± 62.75	26.32 ± 62.26	0.858
T (nmol/L)	3.01 ± 7.10	2.85 ± 8.34	0.891
E2 (HCG day)(pmol/L)	12530 ± 4968	12330 ± 5567	0.790
LH (HCG day)(IU/L)	2.77 ± 3.05	3.25 ± 3.89	0.340
P (HCG day)(nmol/L)	4.31 ± 2.79	4.54 ± 2.83	0.563

OPU, Ovum pick-up; BMI, Body mass index; FSH, Follicle-stimulating hormone; LH, Luteinizing hormone; E_2_, Estradiol; P, Progesterone; PRL, Prolactin; T, Testosterone; HCG, Human chorionic gonadotropin. Measurement data are presented as means ± standard deviations (SD); comparisons between two groups were performed using the t-test. Enumeration data were reported as percentages and compared by the χ^2^ test.

### Stimulation Regimens

In this study, the patients received a long or short regimen used routinely in our center for ovarian induction. For the detailed process, it has been described in our previous literature (16). When 1-2 dominant follicles with a diameter of 18 mm, or more than 2 dominant follicles with a diameter of 17 mm were monitored in the ovaries through a trans-vaginal B-ultrasound, the patient was administered about 5000-10,000 IU of hCG (Lizhu Pharmaceutical Trading Co., Ltd. China). After 24–36 h, OPU was performed to retrieve oocyte corona cumulus complexes (OCCCs).

### Insemination

Under a microscope, the OCCCs in follicular fluid were picked up for 4-6 h of culture *in vitro* in fertilization medium (COOK, USA) and then underwent ICSI or IVF insemination based on each patient’s sperm quality. For ICSI insemination, its detailed process was described in our previously published literature (17), following ICSI, the inseminated oocytes resumed an embryo culture *in vitro* in a cleavage medium (COOK, USA). For IVF insemination, the OCCCs were placed into a fertilization medium containing 5×10^5^/ml grade A and grade B sperm for 5 h of culture *in vitro*. Subsequently, the cumulus cells around the oocytes were removed by a denuding pipette, and their fertilization and ZP were identified. At this time, if the denuded oocytes were found presenting agar-like changes in the ZP, ICSI of the oocytes was performed immediately for rescue insemination followed by embryo culture *in vitro*, this process was defined as an R-ICSI regimen.

### Embryo Culture, Embryo Transfer, and Pregnancy Determination

**Figure 1:**
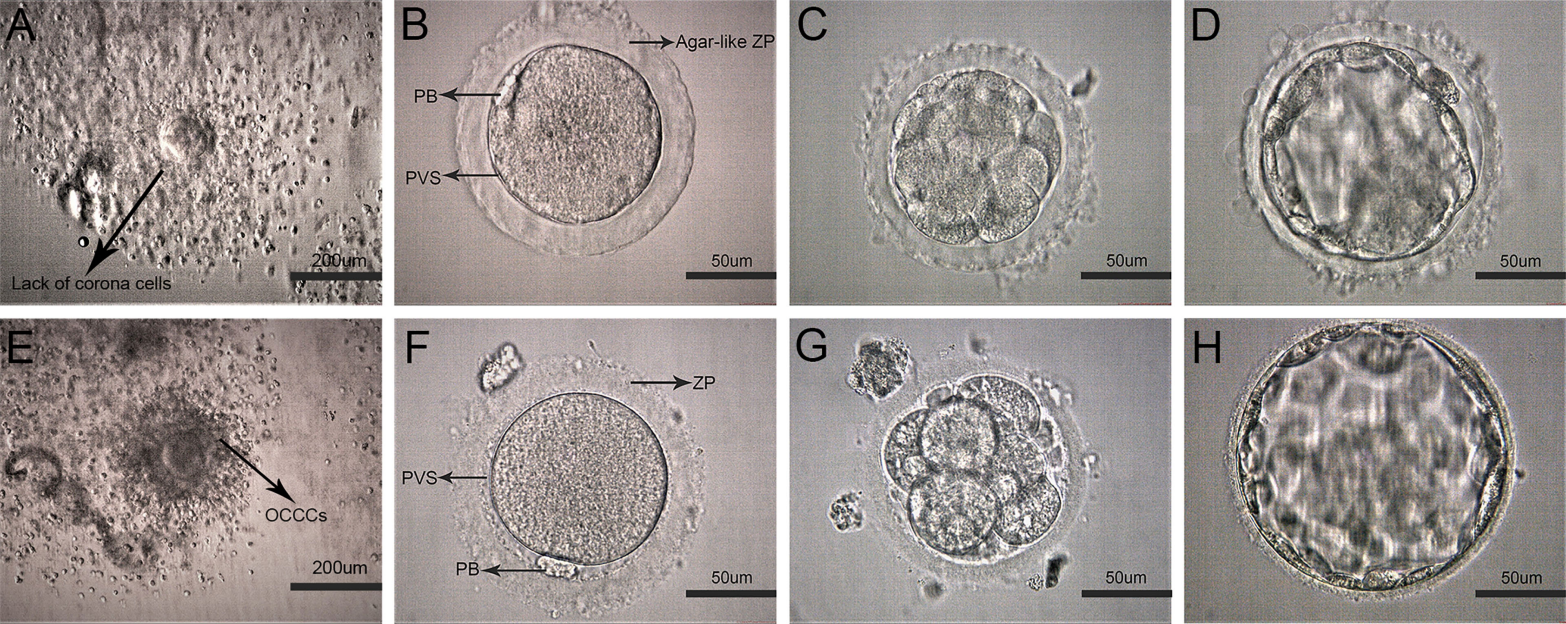
Representative images of early development on human oocytes with agar-like and normal ZP. **(A)** Oocyte corona cumulus complexes (OCCCs) with agar-like ZP (Arrow displayed no obvious corona). **(E)** Normal OCCCs (Arrow displayed clear corona). **(B)** Human mature metaphase II oocyte (MII) with agar-like ZP. **(C**, **D)** Cleavage embryo (D3) and blastocyst(D5) with agar-like ZP. **(F)** MII oocyte with normal ZP. **(G, H)** Cleavage embryo (D3) and blastocyst (D5) with normal ZP.

At 14-18 hours after ICSI/R-ICSI insemination, the fertilized oocytes were selected and continued two days of cleavage embryo culture *in vitro* in cleavage medium (COOK, USA) and a subsequent two or three days of blastocyst culture *in vitro* in blastocyst medium (COOK, USA). Ultimately, the formed high-quality blastocysts (**Figure 1H**) were selected for a fresh embryo transfer or were cryopreserved in -196°C liquid nitrogen through the vitrification method for a following thawed embryo transfer. The processes of embryo culture, vitrification, and embryo transfer have been detailed described in the previously published literature (18). Normally, two weeks after embryo transfer, serum hCG levels were examined and a biochemical pregnancy was confirmed as a positive value of β-hCG (β-hCG ≥ 10 IU/ml) in the blood (19). At 30 days after embryo transfer, the presence of a gestational sac identified by an ultrasound scan was referred to as a clinical pregnancy. In addition, termination of pregnancy at <28 weeks or fetal weight <1 kg is referred to as abortion (20).

### Statistical Analysis

All data analyses were performed using GraphPad Prism 8.0 software (GraphPad Software, San Diego, USA). Descriptive statistics were expressed as the mean ± standard deviation for continuous measures and percentages for enumeration data. For the measurement data, the t-test was adopted, while the χ2 test was used to enumeration data. The results with p-values less than 0.05 (p <0.05) were considered statistically significant.

## Results

### Basic Clinical Information

Body mass index (BMI), basal hormone levels, and hormone levels on HCG day were similar in both groups (**Table 1**). The patients of the case group were generally younger than those of the control group (p<0.05). However, their infertility periods were significantly longer than those in the control group (p<0.05). In this study, 42 patients (72.41%, 42/58) were diagnosed with primary infertility, of which 28 patients (48.28%, 28/58) with a history of pelvic inflammatory disease (PID), and only 10 (17.24%, 10/58) patients with PID in the other 16 infertile patients who were diagnosed as secondary infertility (**Figure S1**).

### The Characteristics of Oocytes With Agar-Like ZP

The characteristics of oocytes with agar-like ZP were shown in **Figure 1**. Before denudation, these OCCCs (**Figure 1A**) did not show obvious corona cells compared with normal OCCCs (**Figure 1E**) through observation under a stereomicroscope; after denudation, the oocytes presented dense and transparent ZP like agar, narrow perivitelline spaces, and flat polar bodies close to the cytoplasm (**Figure 1B**) which were distinctly different from the normal oocytes (**Figure 1F**). Moreover, the ZP of these oocytes showed poor elasticity, high brittleness, and no resistance during the microinjection and maintained agar-like characteristics without obvious expansion at the cleavage (**Figure 1C**) or blastocyst stage (**Figure 1D**) compared to these periods in the normal oocytes (**Figures 1G , H**).

### The Overall Results of Early Embryo Development & Transfer

The detailed data on early embryo development and clinical pregnancy outcomes were shown in **Table 2**. In the case group, a total of 801 oocytes were retrieved in 68 OPU cycles, while in the control group 47557 oocytes were collected in 4156 OPU cycles. The fertilization rate of the case group was significantly higher than that of the control group (P<0.05), while the rates of cleavage, high-quality embryo, blastocyst, and high-quality blastocyst in the case group were greatly lower than those in the control group (all P<0.05). There were 74 embryo transfer cycles in the case group and 5332 cycles in the control group. The rates of cumulative clinical pregnancy and live birth were slightly lower than those in the control group, but there was no significant difference between the two groups. In addition, in the subsequent follow-up, it was found that 41 out of 74 transfer cycles achieved clinical pregnancies, and 34 babies were born successfully. All of the newborns were with no neonatal defects and ≥ 9 average Apgar score (**Table S2**).

**Table 2:** The results of early development and clinical treatment in the patients’ oocytes with agar-like and normal ZP.

Index	Case Group	Control Group	P-value
Fertility rate (%)	82.46 (550/667)	78.03 (37110/47557)	0.0061
Cleavage rate (%)	94 (517/550)	98.58 (36582/37110)	<0.0001
High-quality embryo rate (%)	37.91 (196/517)	44.08 (16125/36582)	0.005
Blastocyst formation rate (%)	49.32 (255/517)	54.51 (19942/36582)	0.0186
High-quality blastocyst formation rate (%)	31.91 (165/517)	44.36 (16228/36582)	<0.0001
Cumulative clinical pregnancy rate (%)	70.69 (41/58)	74.73 (2889/3866)	0.4827
Cumulative holding rate of baby (%)	58.62 (34/58)	59.80 (2312/3866)	0.8553

High-quality embryo: 7–9 blastomeres at equal size on day 3, with no fragmentation or less than 15%. High-quality blastocyst: embryos that were ≥3BB on day 5 or ≥4BB on day 6 by the blastocyst grading system according to Gardner’s criteria. Enumeration data were reported as percentages and compared by the χ2 test.

### The Analysis of ICSI/R-ICSI Intervention on the Clinical Effects of Oocytes With Agar-Like ZP

To explore the ICSI or R-ICSI intervention on clinical effects of these special oocytes, early embryo development & embryo transfer in the ICSI and R-ICSI treatment cycles were further analyzed. In this study, 32 cycles carried out direct ICSI treatment due to poor sperm quality or a previous failed IVF cycle (ICSI group), and another 36 cycles of patients underwent R-ICSI treatment because of an IVF fertilization failure (R-ICSI group). For the rate of fertilization, the R-ICSI group was significantly lower than the ICSI group, with a significant difference between the two groups (p<0.05), while for the rates of cleavage, high-quality embryo, blastocyst, high-quality blastocyst, and the clinical pregnancy and live birth after embryo transfer, the two groups were equivalent, and the detailed data were shown in **Table 3**. No significant difference was found in the baseline data between the two groups, as listed in **Table S1**.

**Table 3:** Embryological characteristics and clinical results of different insemination methods in the case group.

Index	ICSI	R-ICSI	P-value
Total OPU cycles (n)	32	36	–
Transferred cycles (n)	34	40	–
Age(years)	30.63 ± 4.04	29.75 ± 3.83	0.363
Retrieved oocytes (n)	11.22 ± 7.98	12.28 ± 6.25	0.542
Fertilization rate (%)	86.25 (251/291)	79.52 (299/376)	0.023
Cleavage rate (%)	95.62 (240/251)	92.64 (277/299)	0.143
High-quality embryo rate (%)	34.17 (82/240)	41.16 (114/277)	0.102
Blastocyst formation rate (%)	45.83 (110/240)	52.35 (145/277)	0.139
High-quality blastocyst formation rate (%)	29.17 (70/240)	34.3 (95/277)	0.212
Clinical pregnancy rate (%)	50 (17/34)	60 (24/40)	0.388
Live birth rate (%)	38.24 (13/34)	52.5 (21/40)	0.210

ICSI, intracytoplasmic sperm injection; R-ICSI, Early remedial intracytoplasmic sperm injection. Measurement data are presented as means ± standard deviations (SD); comparisons between two groups were performed using the t-test. Enumeration data were reported as percentages and compared by the χ^2^ test.

## Discussion

In this study, the clinical characteristics of patients with ZP agar-like changes in the oocytes were systematically analyzed, and it was found that their basic clinical information was similar to that of the patients with normal oocytes. Hereby, it is difficult to determine whether the patient’s oocytes have undergone agar-like changes. Such special oocytes can only be confirmed after the patient enters IVF treatment and the granulosa cells of oocytes are removed. Normally, such oocytes cannot be fertilized by IVF. Therefore, fertilization disorder is the root cause of infertility in such patients, which is consistent with previous research on other types of abnormal ZP (8, 11, 15, 21, 22).

R-ICSI is a rescue method when the oocytes missed their optimal insemination time. The agar-like ZP oocytes after IVF fertilization failure will continue to perform R-ICSI insemination and subsequent embryo culture *in vitro*. Does this delayed insemination action exert a negative impact on the results of oocyte fertilization and subsequent embryo development? Based on this problem, the ICSI or R-ICSI treatment effects of the patients with the agar-like ZP oocytes were compared and analyzed. The results showed that the fertilization rate of the R-ICSI group was significantly lower than that of the ICSI group, and there was a significant difference between the two groups (p<0.05). However, it was no statistical difference for the cleavage rate, the high-quality embryo rate, the blastocyst rate, the high-quality blastocyst rate, as well as the rates of clinical pregnancy and live birth. The results above indicated that R-ICSI greatly reduced the fertilization rate because of missing the optimal insemination time for the oocytes, but this delay did not seem to affect the subsequent embryo development and clinical treatment effects. The fertilization failure will definitely cause some oocytes to be wasted. If ZP abnormality can be distinguished before ART therapy cycles, it is possible that the ICSI regimen can be performed directly in the subsequent treatment for such patients. On the one hand, it can enhance the utilization rate of oocytes, on the other hand, also improve the overall clinical treatment effects. Therefore, a lot of discussion about the cause of this abnormality has been triggered.

The abnormal ZP observed during IVF/ICSI treatment may be related to external factors, such as stimulation regimens (23), or internal factors, including genetic molecular defects (24), age, and related hormone levels in the body (25). All 20 cycles in 10 patients in this study repeated consistent agar-like ZP despite different ovulation induction regimens in different cycles. We inferred that ZP agar-like changes in oocytes are not related to ovulation induction regimens, more likely attributed to the patient’s own factors. To further evaluate the baseline index between the two groups, BMI and hormone levels related to ovarian function were not different, the age in the case group was younger than control. It might be related to the fact that the majority of patients with agar-like ZP had primary infertility and entered infertility treatment as early as possible. Therefore, ZP agar-like changes in oocytes do not seem to be related to age and hormones. In addition, Occupation and living environment may have an impact on fertility in women, while the telephone follow-up of the patients revealed that the professional background of these patients was not similar, including teachers, nurses, textile workers, construction workers, housewives, etc.; the distribution of living environments varied, from mountainous areas to water towns, and from rural towns to urban areas. There was no special occupational tendency or obvious regional assembled distribution of these objectives in this study. Hereby, it is difficult to speculate the direct disease-causing risk of occupation and living environment in these patients with agar-like ZP oocytes.

Laboratory observations revealed that sperm could bind to the agar-like ZP, but could not penetrate the ZP into the cytoplasm to complete fertilization. Such oocytes showed poor elasticity and high brittleness of ZP during the microinjection. It is speculated the protein structure of agar-like ZP might have been altered. Deletion or mutation of the ZP gene is the most common cause of abnormal structure and function. For example, female mice lacking ZP2 or ZP3 failed to assemble ZP during oocyte growth, which in turn triggered oocyte and ovulation loss, thereby leading to infertility (26, 27). ZP1-deficient mice developed thin-loose ZP and caused early embryo loss and low fertility (28). Recent studies have shown that several mutations in ZP1, ZP2, and ZP3 cause abnormal ZP formation (14, 24, 29). Based on the above findings, the occurrence of agar-like ZP may be associated with the abnormal expression of the ZP gene, but there are few related reports.

It is known that there is no ZP outside the oocyte in the primordial follicle, and only after the start of follicular recruitment, the oocyte, and cumulus cells begin to synthesize ZP glycoprotein together, and this process always occurs in the follicular fluid (30). Follicular fluid is the product of the transfer of plasma components through the blood-follicle barrier, and it contains various substances secreted during the development of oocytes and cumulus cells, which maintains a vital microenvironment for oocyte development (31) and shows different metabolic characteristics at different stages of follicular development (32). In this clinical practice, it was found that the oocytes with agar-like ZP were sparsely surrounded by closely-adherent cumulus cells compared with those of normal patients. This alteration usually reflects the decline of the function and quality of oocytes or an adverse follicular environment, which may lead to the occurrence of abnormal ZP. In the current research, it is noticed that all 38 patients (65.51%, 38/58) had a history of PID. It has been reported that proteins are significantly denatured during inflammation (33). It is possible that ZP agar-like changes in oocytes may be suffered from the attack of inflammatory factors.

At present, the specific pathogenesis of agar-like ZP remains unclear, and there is still a long way to go to establish an effective treatment strategy by elucidating the cause of ZP abnormality. Encouragingly, in this study, a significant difference was found in the structure of OCCCs between the case group and the control group under a stereo microscope, with almost no obvious coronary cells observed in the former. This discovery can provide an effective basis for timely adjustment of fertilization strategy for agar-like ZP patients undergoing IVF treatment. Previous studies reported that although ICSI fertilization was able to achieve pregnancies and live births in the patients with ZP abnormalities, pregnancy outcomes were still not ideal (8, 15). To further illuminate the effects of agar-like ZP on the potential of embryo development, the embryological characteristics and clinical results of the patients with agar-like ZP and those with normal ZP (excluding malefactors) were compared and analyzed in this study. The results showed that the fertilization rate of the case group was significantly higher, while the cleavage rate, the high-quality embryo rate, the blastocyst rate, and the high-quality blastocyst rate were significantly lower than control, indicating that the agar-like ZP severely weakened the oocyte’s development competence. As for a better fertilization outcome in the case group, it might be related to the use of ICSI insemination. After clinical embryo transfer, the rates of clinical cumulative pregnancy and live birth in the case group were comparable to those in the control group. In the subsequent follow-up, it was found that 34 of the 58 patients in the present study gave birth to babies. All of the newborns were healthy with no neonatal defects and ≥ 9 average Apgar score. The above results indicate that although the development potential of oocytes with agar-like ZP is lower than normal oocytes, there are still a certain number of oocytes that can develop normally and eventually deliver healthy offspring.

In summary, the pathogenesis of oocytes with agar-like ZP remains unclear. So far, such patients cannot be diagnosed in advance based on specific clinical characteristics. Insemination disorder is the root cause of infertility in such patients. Both R-ICSI and direct ICSI regimens can allow the oocytes to achieve normal fertilization, normal embryo development as well as healthy offspring, in which direct ICSI regimen can bring better clinical treatment effects. Moreover, the distinct structure of OCCCs seems to provide an effective basis for timely adjustment of insemination strategy for the oocytes with agar-like ZP.

In conclusion, the occurrence of agar-like ZP impairs the development competence of human oocytes, however, the human oocytes with agar-like ZP can develop into healthy offspring, and an ICSI regimen is the optimal treatment strategy for them.

## Data Availability Statement

The original contributions presented in the study are included in the article/**Supplementary Material**. Further inquiries can be directed to the corresponding authors.

## Ethics Statement

The studies involving human participants were reviewed and approved by Ethics Committee of Anhui Medical University. The patients/participants provided their written informed consent to participate in this study.

## Author Contributions

ZZ, BC, and WZ designed the experiments. DY, HY, and BY performed the experiment and wrote the manuscript. KW and QZ collected data from patients. JW and FD collated patients’ data. BR and RX analyzed the statistical data. JP and QW reviewed the data statistic. YC revised the manuscript. All authors contributed to the article and approved the submitted version.

## Funding

This study was supported by the Youth Development Programme of the First Affiliated Hospital of Anhui Medical University, 2018 (No.2849), National Natural Science Foundation of China (No.82071724, 82001516&82001635), and an Open project of State Key Laboratory of Reproductive Medicine (No. SKLRM-K202005).

## Conflict of Interest

The authors declare that the research was conducted in the absence of any commercial or financial relationships that could be construed as a potential conflict of interest.

## Publisher’s Note

All claims expressed in this article are solely those of the authors and do not necessarily represent those of their affiliated organizations, or those of the publisher, the editors and the reviewers. Any product that may be evaluated in this article, or claim that may be made by its manufacturer, is not guaranteed or endorsed by the publisher.
