# Supplementary material for: Embryological Characteristics of Human Oocytes With Agar-Like Zona Pellucida and Its Clinical Treatment Strategy
Source: Front Endocrinol (Lausanne). 2022 Jun 23;13:859361. doi: 10.3389/fendo.2022.859361 (PMC9259955; doi:10.3389/fendo.2022.859361)

## Supplementary Figures


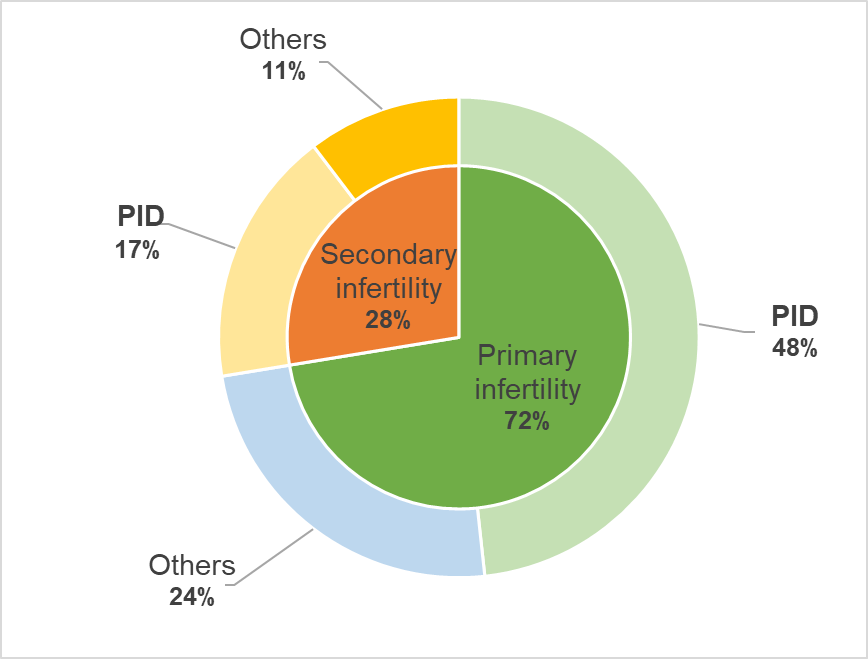


**Figure.S1** **Causes of 58 infertile patients with agar-like ZP (Female factors).** Causes of 58 infertile patients with agar-like ZP (Female factors). 42 patients (72.41%, 42/58) were diagnosed with primary infertility, of which 28 patients (48.28%, 28/58) with a history of pelvic inflammatory disease (PID), and only 10 (17.24%, 10/58) patients with PID in other 16 infertile patients who diagnosed as secondary infertility.

## Supplementary Tables

**Table.S1 Comparison of basic clinical information of the patients between the ICSI and the R-ICSI groups**

| **Index** | **ICSI** | **R-ICSI** | **P-value** |
| --- | --- | --- | --- |
| Total OPU cycles (n)  Transferred cycles (n) | 32  34 | 36  40 | -  - |
| Age(years) | 30.63±4.04 | 29.75±3.83 | 0.363 |
| Infertility duration(years)  Retrieved oocytes (n) | 4.65±3.71  11.22±7.98 | 4.26±2.65  12.28±6.25 | 0.638  0.542 |
| BMI (kg/m^2^) | 22.65±4.17 | 22.02±3.29 | 0.542 |
| FSH (IU/L) | 7.79±2.76 | 7.13±2.20 | 0.347 |
| LH (IU/L) | 4.62±3.13 | 4.77±2.11 | 0.842 |
| E2 (pmol/L) | 160.7±104.9 | 182.4±134.1 | 0.523 |
| P (nmol/L) | 2.20±1.53 | 2.18±2.14 | 0.981 |
| PRL (ng/ml) | 13.73±3.72 | 12.61±6.43 | 0.678 |
| T (nmol/L) | 1.56±0.64 | 1.35±0.60 | 0.251 |
| E2 (HCG day) (pmol/L) | 12097±4395 | 12577±5342 | 0.726 |
| LH (HCG day) (IU/L) | 2.20±1.21 | 1.66±1.12 | 0.109 |
| P (HCG day) (ng/ml) | 4.45±2.98 | 4.19±2.71 | 0.748 |

ICSI: intracytoplasmic sperm injection; R-ICSI: Early remedial intracytoplasmic sperm injection. Measurement data are presented as means ± standard deviations (SD); comparisons between two groups were performed using the t-test. Enumeration data were reported as percentages and compared by the χ^2^ test.

**Table.S2 Details of newborns in the case group**

| **Patients** | **Insemination methods** | **Infant Gender** | **Apgar** | **Body weight (g)** |
| --- | --- | --- | --- | --- |
| 1 | R-ICSI | Male | 10 | 3400 |
| 2 | ICSI | Male | 10 | 2400 |
| 3 | R-ICSI | Male | 10 | 4330 |
| 4 | ICSI | Female | 10 | 3250 |
| 5 | R-ICSI | Male | 10 | 3750 |
| 6 | R-ICSI | Male | 9 | 3100 |
| 7 | ICSI | Male | 10 | 4000 |
| 8 | R-ICSI | Female | 10 | 3200 |
| 9 | R-ICSI | Female | 10 | 3150 |
| 10 | R-ICSI | Male | 10 | 3650 |
| 11 | ICSI | 2Male | 10*2 | 2700*2 |
| 12 | ICSI | Male | 10 | 3700 |
| 13 | ICSI | Male | 10 | 3000 |
| 14 | R-ICSI | Male | 10 | 3320 |
| 15 | ICSI | Male | 9 | 3300 |
| 16 | ICSI | Female | 10 | 3250 |
| 17 | ICSI | Female | 10 | 4750 |
| 18 | R-ICSI | Male | 10 | 4000 |
| 19 | R-ICSI | Male | 10 | 4005 |
| 20 | R-ICSI | Male/Female | 10*2 | 3100/2800 |
| 21 | R-ICSI | Female | 10 | 3470 |
| 22 | R-ICSI | Female | 10 | 3300 |
| 23 | ICSI | Male | 10 | 4000 |
| 24 | R-ICSI | Male/Female | 10*2 | 2800/2600 |
| 25 | ICSI | 2Male | 10*2 | 2800 |
| 26 | ICSI | Female | 10 | 3600 |
| 27 | R-ICSI | Female | 10 | 3350 |
| 28 | R-ICSI | Male | 10 | 4000 |
| 29 | ICSI | Female | 10 | 3950 |
| 30 | R-ICSI | Male | 10 | 3400 |
| 31 | R-ICSI | Female | 10 | 3750 |
| 32 | R-ICSI | Male | 10 | 2800 |
| 33 | R-ICSI | Male | 10 | 3700 |
| 34 | R-ICSI | Male | 10 | 3900 |

## Microscopic photographs of the oocytes of some patients in the case group

Clinical data of the infertile patients undergoing IVF/ICSI treatment in the First Affiliated Hospital of Anhui Medical University from May 2015 to October 2020 were collected. Unfortunately, we only have pictures of a small number of patients, and most of the patients with agar-like ZP we have only marked in the embryo laboratory record sheet, without keeping pictures of their oocytes.

Case 1


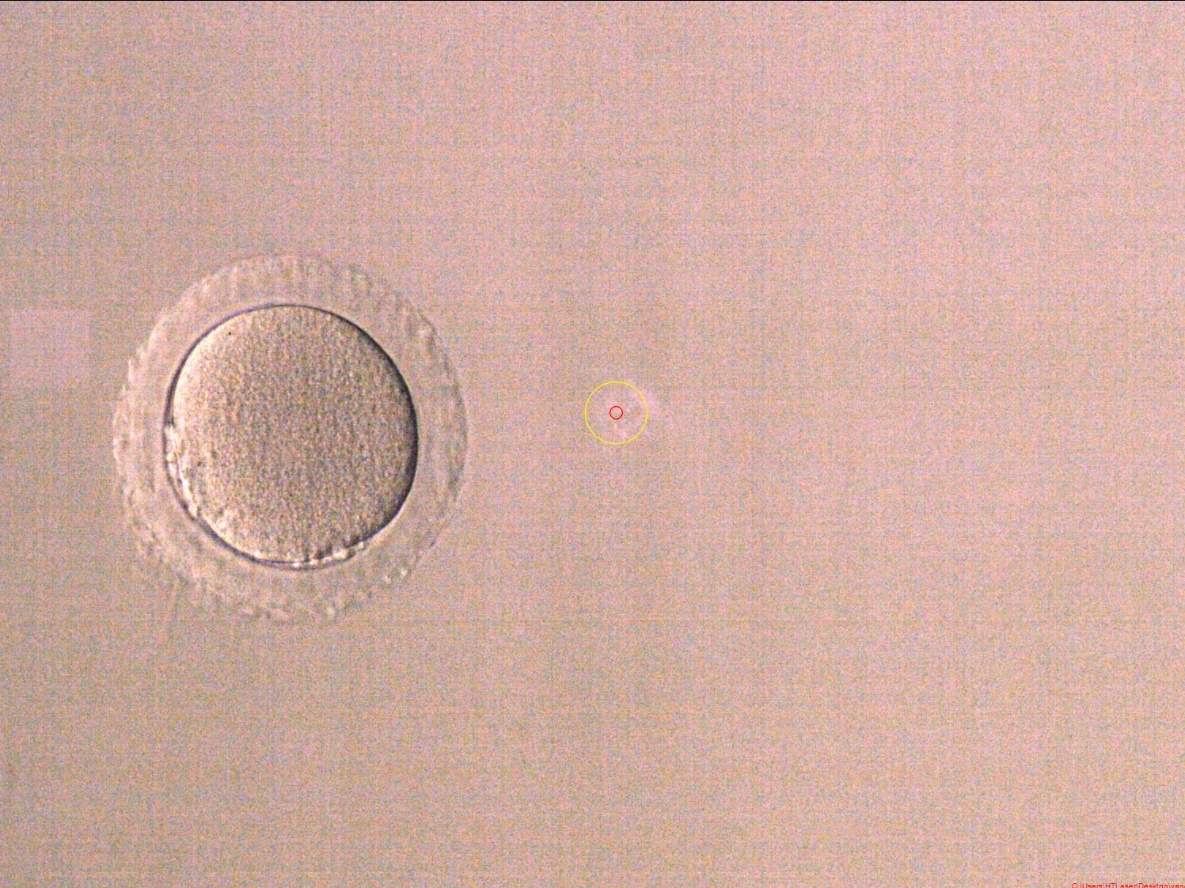


Case 2


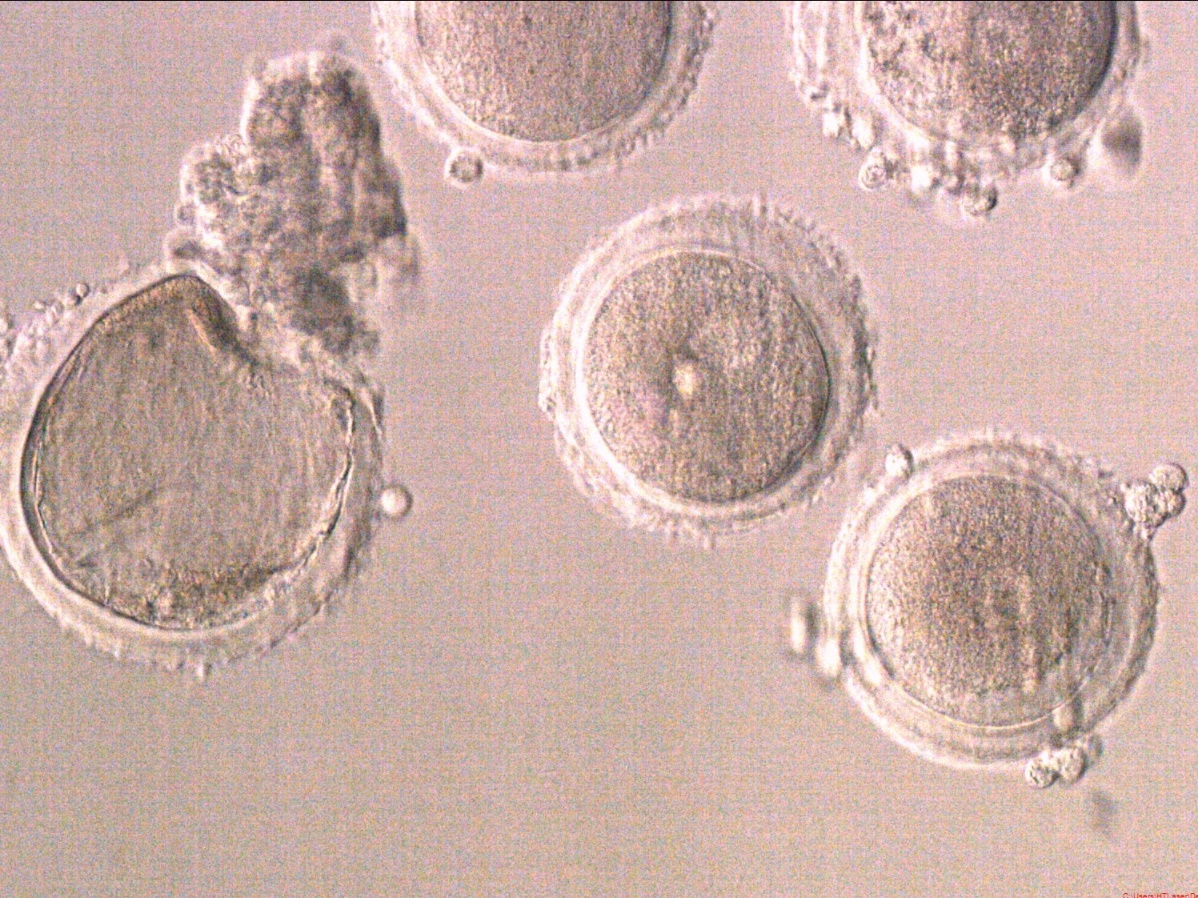


Case 3


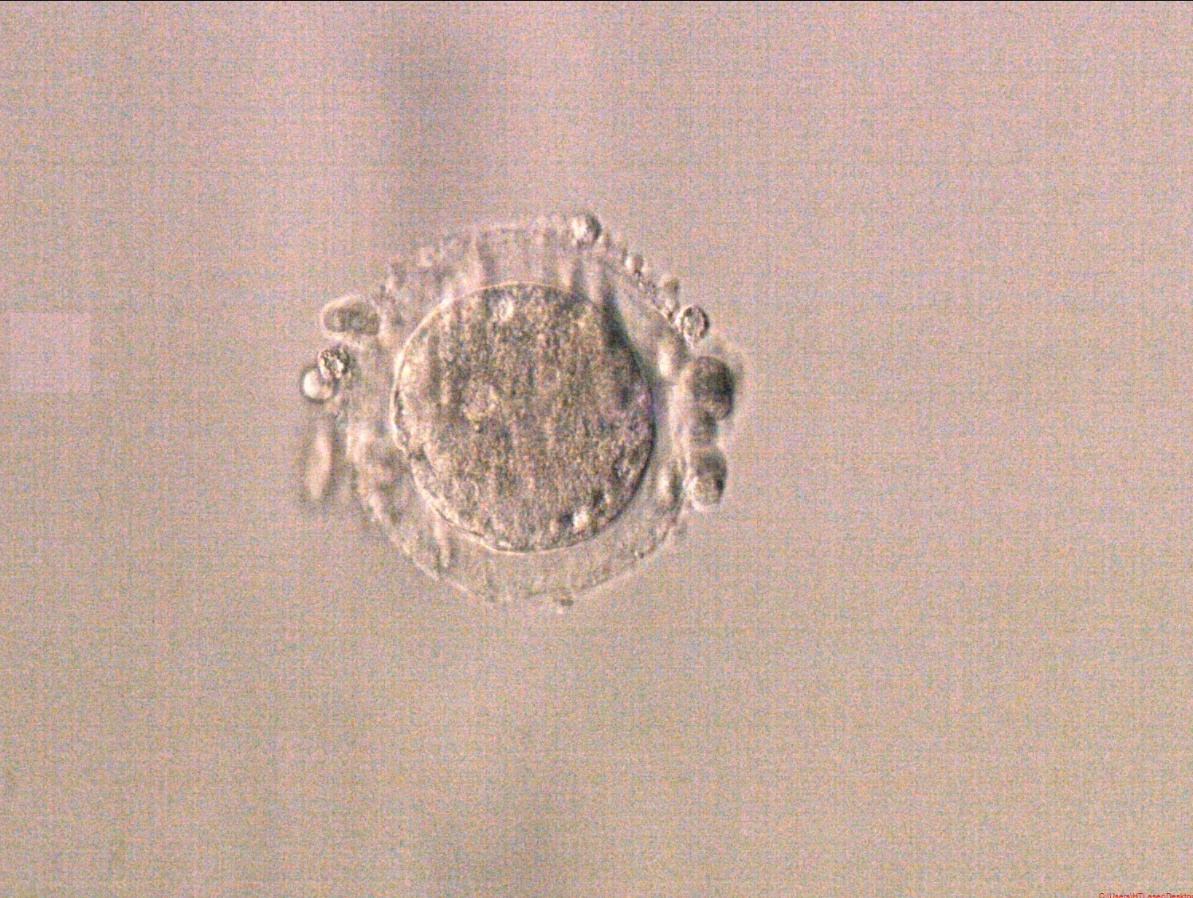


Case 4


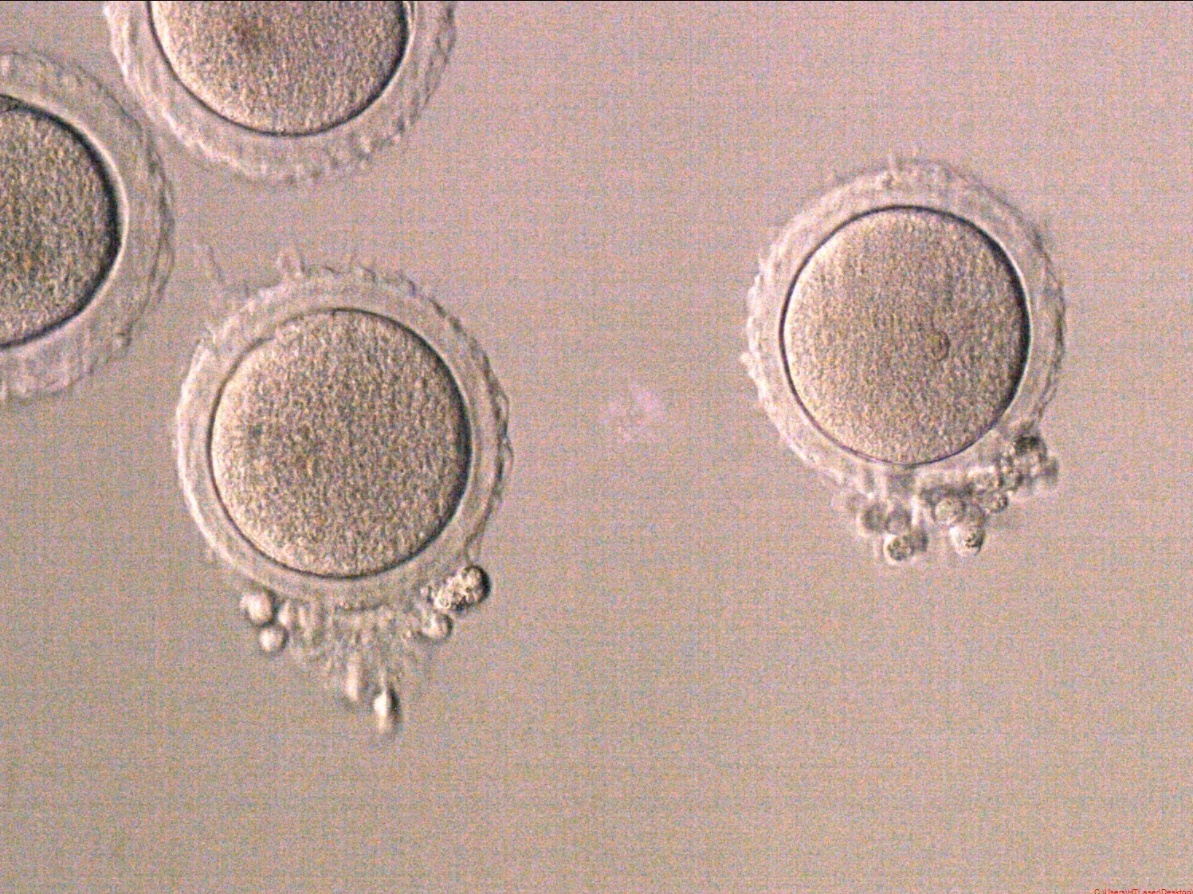


Case 5


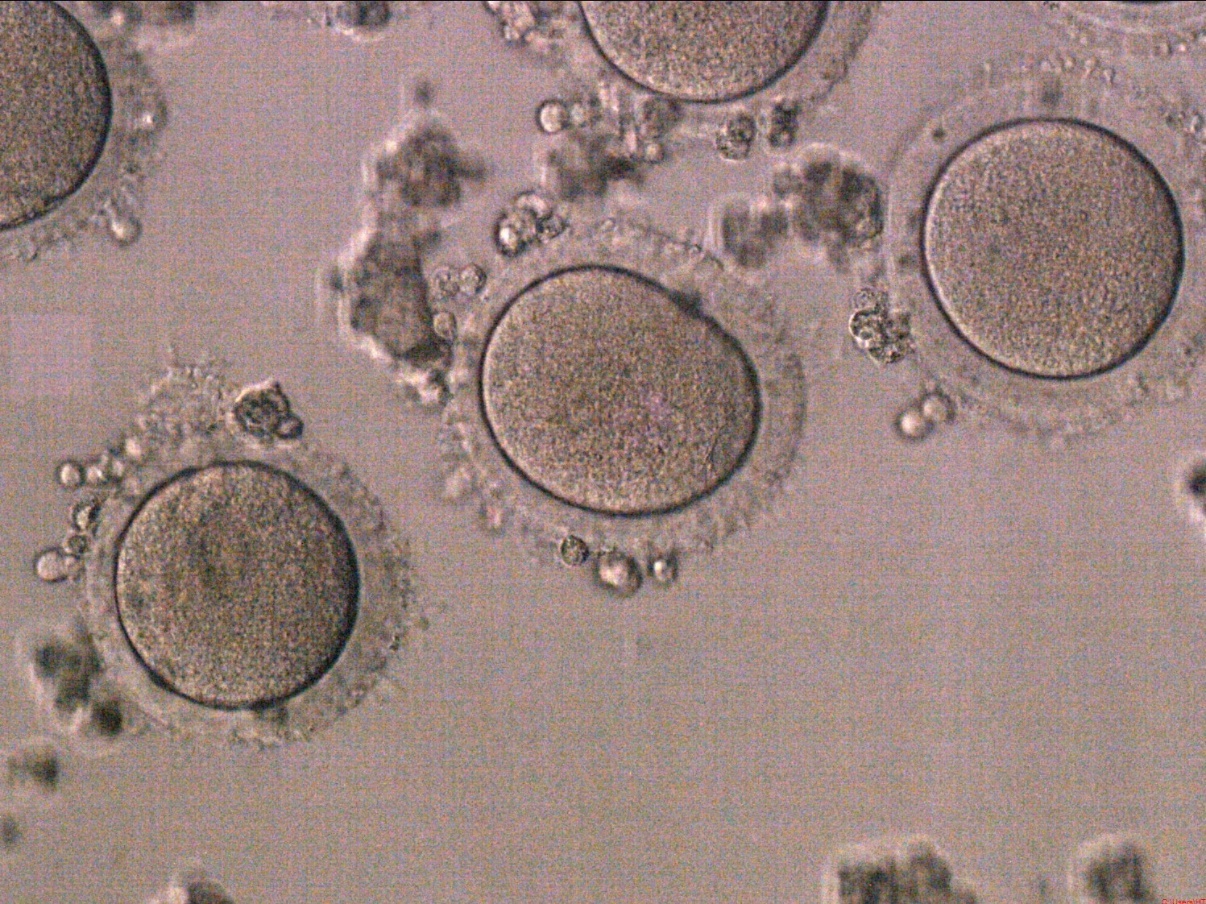

Supplement: Supplementary file 1 [file DataSheet_1.docx]
